# Temporal and Spatial Resolution of Activated Plant Defense Responses in Leaves of *Nicotiana benthamiana* Infected with *Dickeya dadantii*

**DOI:** 10.3389/fpls.2015.01209

**Published:** 2016-01-08

**Authors:** María L. Pérez-Bueno, Espen Granum, Mónica Pineda, Víctor Flors, Pablo Rodriguez-Palenzuela, Emilia López-Solanilla, Matilde Barón

**Affiliations:** ^1^Department of Biochemistry and Molecular and Cell Biology of Plants, Estación Experimental del Zaidín, Spanish Council of Scientific Research (CSIC)Granada, Spain; ^2^Department of Agricultural and Environmental Sciences, Universitat Jaume ICastellón, Spain; ^3^Departamento de Biotecnología, Centro de Biotecnología y Genómica de Plantas, Universidad Politécnica de MadridMadrid, Spain

**Keywords:** *Dickeya dadantii*, necrotroph, plant resistance, chlorophyll fluorescence imaging, multicolor fluorescence imaging, thermal imaging

## Abstract

The necrotrophic bacteria *Dickeya dadantii* is the causal agent of soft-rot disease in a broad range of hosts. The model plant *Nicotiana benthamiana*, commonly used as experimental host for a very broad range of plant pathogens, is susceptible to infection by *D. dadantii.* The inoculation with *D. dadantii* at high dose seems to overcome the plant defense capacity, inducing maceration and death of the tissue, although restricted to the infiltrated area. By contrast, the output of the defense response to low dose inoculation is inhibition of maceration and limitation in the growth, or even eradication, of bacteria. Responses of tissue invaded by bacteria (neighboring the infiltrated areas after 2–3 days post-inoculation) included: (i) inhibition of photosynthesis in terms of photosystem II efficiency; (ii) activation of energy dissipation as non-photochemical quenching in photosystem II, which is related to the activation of plant defense mechanisms; and (iii) accumulation of secondary metabolites in cell walls of the epidermis (lignins) and the apoplast of the mesophyll (phytoalexins). Infiltrated tissues showed an increase in the content of the main hormones regulating stress responses, including abscisic acid, jasmonic acid, and salicylic acid. We propose a mechanism involving the three hormones by which *N. benthamiana* could activate an efficient defense response against *D. dadantii*.

## Introduction

The necrotrophic bacteria *Dickeya* sp. and *Pectobacterium* sp. are the causal agents of the soft-rot disease in a broad range of hosts, affecting one-half of angiosperm plant orders (Ma et al., 2007). As a result, they cause great economic losses in crops and ornamental plants world-wide (Reverchon and Nasser, 2013). *Dickeya dadantii* strain 3937, formerly known as *Erwinia chrysanthemi* 3937, was originally isolated from African violet (*Saintpaulia ionantha*). This necrotrophic bacterium is especially pernicious due to its ability to cause latent infections, which become active in post-harvest, affecting the shelf life of the product. Furthermore, *D. dadantii* can survive as saprophyte, epiphyte or endophyte, being a frequent inhabitant of leaves (Haygood et al., 1982), inland waters (Cother and Gilbert, 1990) and soils (Burr and Schroth, 1977). In consequence, *D. dadantii* is widely distributed and persistent in agronomic ecosystems worldwide. Particularly in Southern Europe, *D. dadantii* has been identified as an emergent problem (Palacio-Bielsa et al., 2010).

Necrotrophic pathogens have developed a wide range of virulence strategies, including the secretion of phytotoxic compounds and cell wall-degrading enzymes, to promote cell death and leakage of nutrients to feed on. In some cases, the plant immune system is effective against the necrotroph, restricting further ingress and disease symptoms development (Mengiste, 2012). The pathogenesis of *D. dadantii* has been intensively studied at the molecular level during the last decades. The traditional approach has emphasized the role of multiple exozymes (including pectinases, cellulases, and proteases) producing maceration of the infected tissue by breaking down plant cell walls, as reviewed by Toth et al. (2003).

Many authors have addressed the effect of biotrophs and hemi-biotrophs on plant physiology. However, photosynthetic responses in plants to necrotrophs have been paid little attention. A recent study (Göhre et al., 2012) demonstrated how PAMPs triggered immunity response, which influences photosynthesis and their crosstalk via the NPQ on *Arabidopsis* plants treated with the PAMP flg22, a peptide derived from flagellin. NPQ is a defense mechanism related to photosynthesis that protects the thylakoid membrane of the chloroplast from excess excitation energy by safely dissipating it. NPQ plays a crucial role in plant fitness and under any stress condition that could compromise or inhibit the activity of the thylakoid electron transport chain, expressed in terms of quantum yield of photosystem II (Φ_PSII_). In previous studies on *Nicotiana benthamiana* plants infected with pepper mild mottle virus (Pérez-Bueno et al., 2006) as well as on *Phaseolus vulgaris* infected with *Pseudomonas syringae* pv. *phaseolicola* (Rodríguez-Moreno et al., 2008; Pérez-Bueno et al., 2015), we demonstrated that NPQ plays an important role in plant defense against pathogens. Inhibition of photosynthesis upon infection could divert the major flow of carbon from primary metabolism to secondary metabolism for the biosynthesis of phenolic compounds (Bolton, 2009; Barón et al., 2012). These phenolic compounds can act as physical and chemical barriers against infection (for a review see Dixon et al., 2002).

Another plant physiological process playing an important role in pathogen infections is the stomatal function. A recent review by Sawinski et al. (2013) highlights the importance of stomatal regulation in innate plant immunity. Stomata are the main natural entry site for pathogens and an activation of stomatal closure upon detection of potentially pathogenic microbes appears as an essential part of the plant defense against pathogens. On the other hand, pathogens are frequently able to manipulate stomata regulation and activate opening of guard cells. Stomatal aperture controls the leaf transpiration rate, and therefore determines the leaf temperature, which can easily be analyzed by thermography, as revised by Chaerle and Van Der Straeten (2001).

The aim of the present study is to gain knowledge about the defense responses of *N. benthamiana* elicited by the necrotroph *D. dadantii* 3937. Two fluorescence techniques combined with thermal imaging were employed to obtain spatial and temporal information about stomatal regulation, primary and secondary metabolism throughout the infection and analyze their role in plant defense. This study was complemented by the localization of secondary metabolites within the structure of the leaves by confocal microscopy and a quantitative analysis of secondary metabolites and hormones. Taken all together, these results point to a possible defense mechanism by which *N. benthamiana* activates resistance to *D. dadantii*.

## Materials and Methods

### Biological Material and Inoculation

*Nicotiana benthamiana* plants were grown at 150 μmol m^-2^ s^-1^ photosynthetically active radiation, using white fluorescent lamps (HPI-T 250 W; Phillips, Eindhoven, The Netherlands), with a 16/8 h (22/18°C) light/dark photoperiod and 65% relative humidity. *D. dadantii* 3937 was grown for 24 h at 28°C in Luria-Bertani (LB) plates containing 25 μg ml^-1^ rifampicin.

**FIGURE 1:**
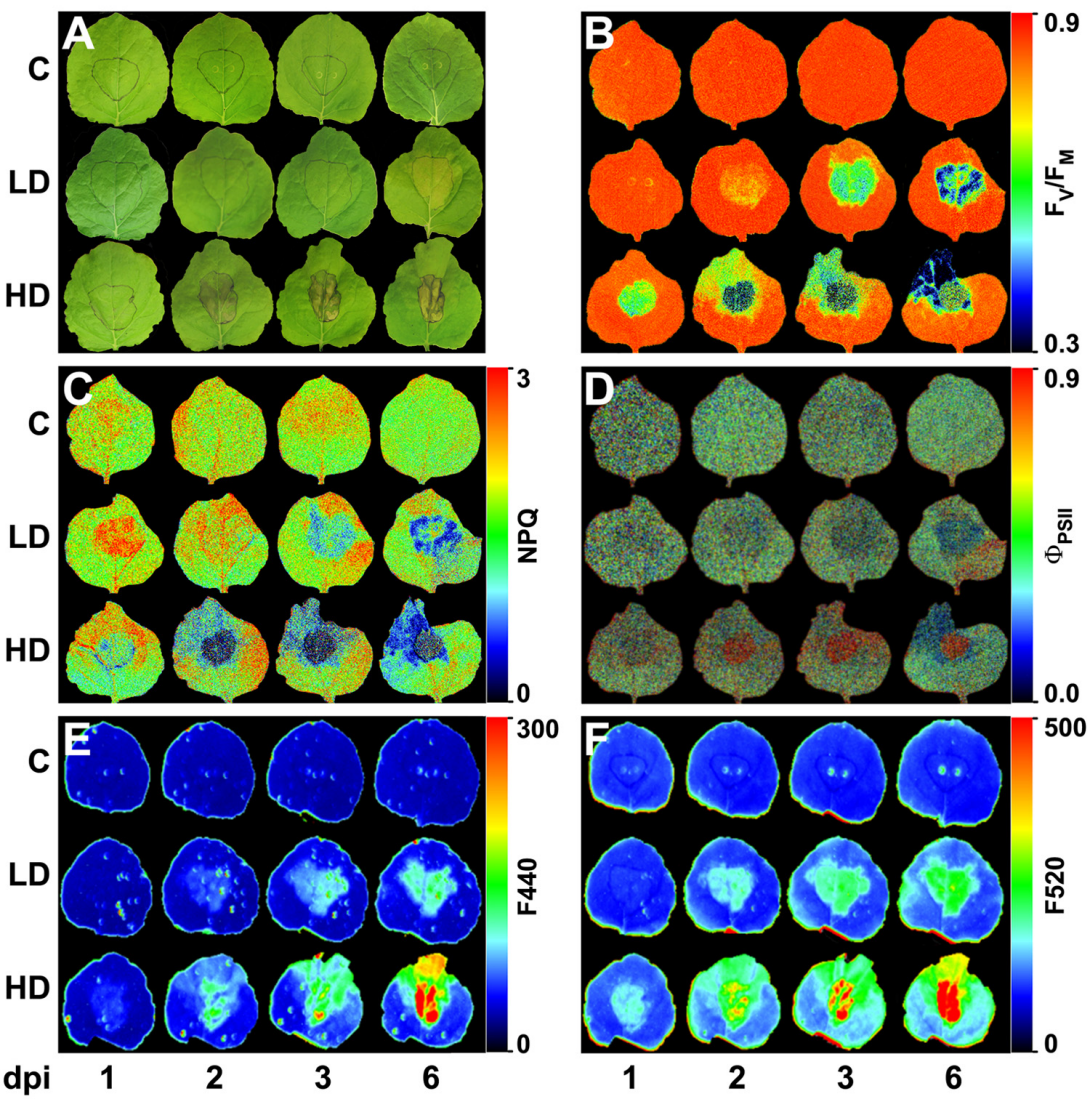
***Nicotiana benthamiana* leaves inoculated with *Dickeya dadantii* at a concentration of 10^4^ (LD) or 10^6^ (HD) cfu per ml or mock-inoculated plants (C)**. Evolution of symptoms **(A)** and images at different post-infection times of: maximum quantum efficiency of PSII **(B)**, NPQ in the light-adapted steady state **(C)**, quantum efficiency of PSII in the light-adapted steady state **(D)**, and fluorescence at 440 nm **(E)**, and 520 nm **(F)**. The infiltrated area was accurately outlined. The false color-scale used in (**B–F)** is depicted for each parameter. Images show a representative measurement.

Fully developed leaves of 4 weeks-old plants were inoculated by pressing the bacterial suspension into the abaxial side of the leaf using the blunt end of a 1 ml syringe, with bacterial suspensions at 10^4^ or 10^6^ cfu per ml in 10 mM MgCl_2_, LD and HD respectively. Mock-inoculated control plants (C) were infiltrated with 10 mM MgCl_2_. The infiltrated area was accurately outlined using a marker pen, as shown in **Figure 1A**. Three regions of the leaf were analyzed: the infiltrated area (I), and the non-infiltrated tip (T) and base (B) of the leaf. All measurements were repeated in four independent experiments.

Determination of bacterial density per leaf area was performed by extracting bacteria from two 0.5 cm^2^ leaf disks ground in 10 mM MgCl_2_. Serial dilutions of the bacteria in 10 mM MgCl_2_ were plated onto LB plates and cfu counts performed after 48 h.

### Photosynthetic Parameters

The photosynthetic activity was evaluated by Chl-F imaging, carried out with an Open FluorCam FC 800-O (PSI, Brno, Czeck Republic) kinetic imaging fluorometer controlled by FluorCam7 (PSI) software. Measuring light flashes (10 μs) for modulated Chl-F excitation were generated by a pair of red LED panels (λ_max_∼618 nm), and saturating light pulses (1 s, ∼2000 μmol m^-2^ s^-1^) and actinic light by a pair of blue LED panels (λ_max_∼455 nm). Chl-F emission kinetics was captured by a B&W CCD camera with 12-bit and 1024 × 768 pixel resolution, taking 10 images per second. Reflected radiation was blocked by a far-red filter (RG697, Schott, Mainz, Germany). Measurements of minimum and maximum fluorescence in the dark-adapted state (F_0_ and F_M_, respectively) and after 20 min light-adaptation (F_t_ and F’_M_) were used to determine maximum quantum yield as F_V_/F_M_ = (F_M_-F_0_)/F_M_, NPQ in the light-adapted steady-state as NPQ = (F_M_-F’_M_)/F’_M_, and quantum efficiency of PSII as ϕ_PSII_ = (F’_M_-F_t_)/F’_M_ (Maxwell and Johnson, 2000). Images were displayed using a false color scale applied by the FluorCam software version 7.1.0.3. Average F_V_/F_M_, NPQ and ϕ_PSII_ values were determined over the area of infiltrated (in the middle of the leaf) and non-infiltrated regions (base and tip) of each leaf. All measurements were carried out on attached leaves at 1, 2, 3, and 6 days post-inoculation for each treatment. Images presented correspond to the most representative data.

### Leaf Thermography

Infrared images of plant leaves were taken in the growth chamber with a Photon 640 camera (FLIR Systems, Wilsonville, OR, USA) vertically positioned approximately 0.5 m above the leaves according to Pérez-Bueno et al. (2015). Immediately after infiltration, three plants representing each treatment were positioned for simultaneous imaging of one leaf from each plant, and images were recorded every 10 min during the first day, and then once a day (at midday) over a period of 6 days. Average temperatures were determined for infiltrated and non-infiltrated regions of each leaf.

### Autofluorescence by Multicolor Fluorescence Imaging

Multicolor fluorescence imaging was performed on control and *D. dadantii* infected plants using an Open FluorCam FC 800-O (Photon Systems Instruments, Brno, Czech Republic). Multicolor fluorescence emission in the blue (F440) and green (F520) regions of the fluorescence spectrum was acquired for attached leaves at 1, 2, 3, and 6 dpi, as described in Pérez-Bueno et al. (2015). The excitation wavelength used was 355 nm. Black and white images of fluorescence were displayed using a false color scale applied by the FluorCam software version 7.1.0.3. Numerical data from the regions of interest were also processed. Four different plants per treatment were analyzed. Images presented correspond to the most representative data.

### Localization of Phenolic Compounds by Confocal Laser Scanning Microscopy

Fresh leaf samples (infiltrated region and leaf tip) were cut out and incubated on microscopic slides in a droplet of water, and autofluorescence of the samples was studied with a Nikon C1 laser scanning confocal microscope (Nikon Instruments Inc., Japan) at an excitation wavelength of 405 nm. CLSM images were obtained for blue (400–430 nm), green (515–565 nm), and red (>650 nm) fluorescence channels at different focal planes (epidermal and mesophyll layers) using a 40× oil immersion objective. To compare autofluorescence in leaves from different treatments at 1–6 dpi all measurements were conducted with equal sensitivity settings. Images presented correspond to the most representative data.

### Determination of Secondary Metabolites

The determination of total soluble phenolic compounds was performed as previously described (Chun and Kim, 2004). A colorimetric reaction using Folin’s reagent (Merck Darmstadt, Germany) at 4.7% on methanol leaf extracts was followed at 765 nm. Caffeic acid was used as standard for the calibration curve.

Secondary metabolites and phytohormones were analyzed by LC-ESI. 50 mg of freeze-dried leaf samples were extracted with MeOH:H_2_O (10:90) containing 0.01% of HCOOH. Before extraction, a mixture of internal standards containing 100 ng dihydrojasmonic acid, d5-ABA, d6-SA, and propyl-paraben was added. After polytron homogenization on ice, the samples were centrifuged for 15 min at 15000 *g* and the supernatant was filtered and an aliquot was used for subsequent analysis. An Acquity ultra-performance liquid chromatography system (UPLC, Waters, Mildford, MA, USA) was interfaced to a triple quadrupole mass spectrometer (TQD, Waters, Manchester, UK). The chromatographic separation conditions were closely related to those described previously. The LC separation was performed by HPLC SunFire C18 analytical column, 5 μm particle size, 2.1 mm × 100 mm (Waters, Mildford, MA, USA). Chromatographic conditions and TQD parameters were followed as described in Agut et al. (2014). Compound quantities were compared with their respective standard curves for ABA, SA, SAG, SGE, chlorogenic acid, ferulic acid, and scopoletin. Quantifications where carried out with Mass Lynx (v 1.4, Mycromass) software using the internal standards as reference for extraction recovery and the standard curves as quantifiers.

### Bacterial Growth Inhibition Assays

The inhibition of bacterial growth by SA and scopoletin (Sigma-Aldrich, St. Louis, MO, USA) was tested in *D. dadantii* suspensions at 10^4^ cfu ml^-1^ in LB liquid medium containing 25 μg ml^-1^ rifampicin and grown for 24 h at 28°C. The optical density at 600 nm was recorded at time 0 and 24 h. The percentage of bacterial growth was calculated referring the optical density of the bacterial suspensions containing increasing concentrations of SA or scopoletin to bacterial suspensions. For each experiment, three replicates per treatment were considered (control and the different SA/scopoletin concentrations tested. The percentage of growth inhibition was calculated as 100^∗^(DO_600-0__mM_ – DO_600__×__mM_)/DO_600-0__mM_. The experiment was repeated three times with identical results. All data from the three experiments were taken in account for the calculation of average and SE values.

### Statistics

All calculations were performed with Microsoft Office Excel 2010 (Microsoft Corporation, Redmond, WA, USA). Statistical analysis of data was carried out using Student’s *t*-test with SigmaPlot (Systat Software, Inc., Richmond, CA, USA).

## Results

### Symptomatology and Bacterial Growth in Infected Plants

The response of *N. benthamiana* to bacterial challenge was analyzed by comparing the effects of inoculation using two bacterial concentrations: the so called LD (10^4^ cfu ml^-1^), considered to be closer to natural infection conditions, and HD (10^6^ cfu ml^-1^).

In LD leaves, infiltrated areas developed a very slight chlorosis at 3 dpi, that spread out of the infiltration site after 6 dpi, as marked in **Figure 1A**. On the contrary, the areas infiltrated with HD showed clear signs of maceration at 1 dpi and necrosis at 2 dpi. Chlorosis appeared at the leaf tip at 3 dpi and spread toward the base of the leaf within the time of study, only in HD leaves (**Figure 1A**).

**FIGURE 2:**
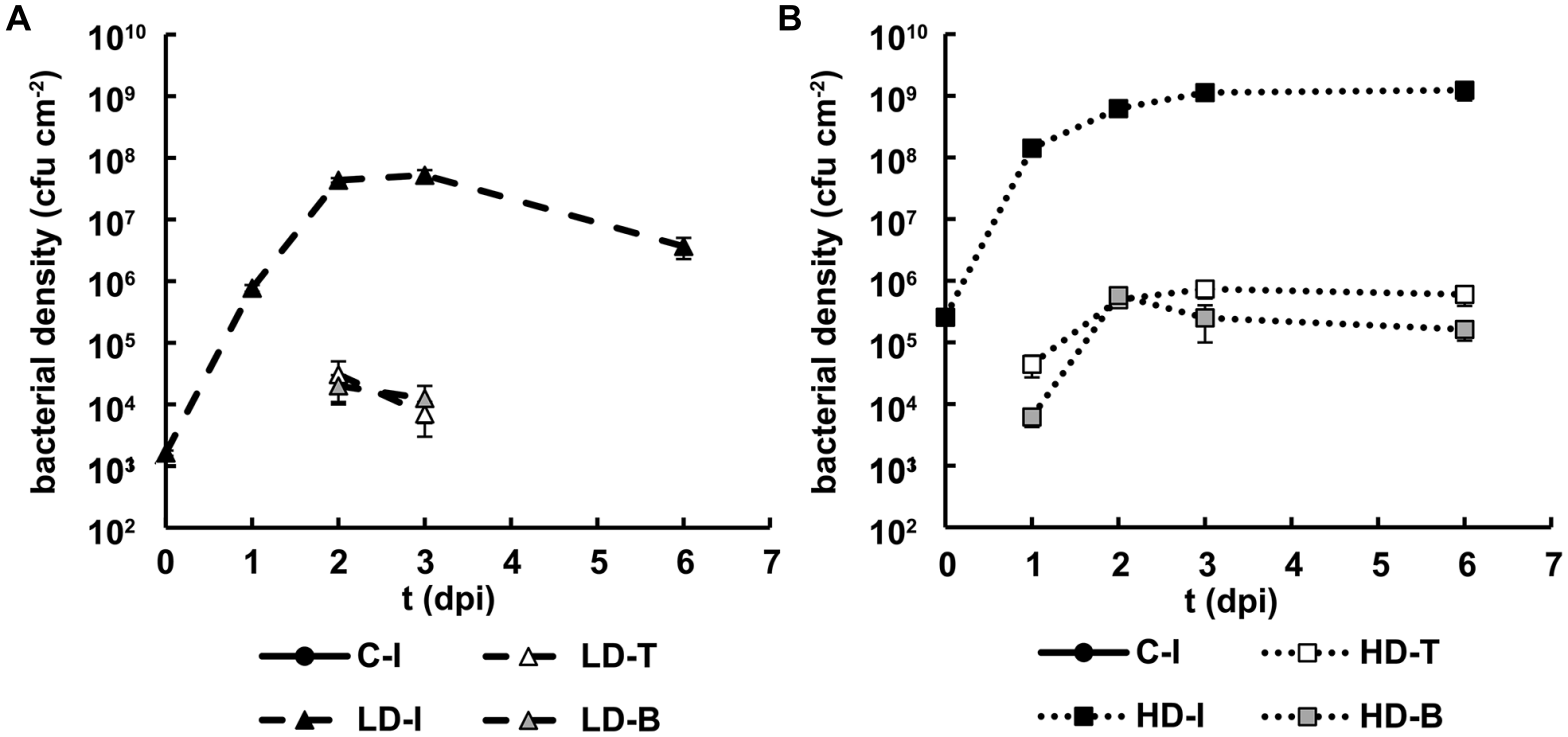
**Bacterial growth in *N. benthamiana* leaves inoculated with *D. dadantii***. Three regions of interest have been analyzed: the infiltrated (I) as well as the tip (T) and base (B) of non-infiltrated areas, surrounding the infiltrated area. Bacterial density found in leaves inoculated at LD **(A)** and HD **(B)**. Error bars indicate standard errors, *n* = 10.

Bacterial density in the infiltrated areas increased up to 10^8^–10^9^ cfu cm^-2^ in 1 dpi for HD and 2 dpi for LD, and then remained stable throughout the period of study (**Figures 2A,B**). In the neighboring regions, tip and base of the leaf, the bacterial density reached ∼10^5^ cfu cm^-2^ at 1 dpi in HD plants and 1 day later in LD leaves. Only in the last case the bacterial density decreased during the later stages of infection. Indeed, no colony-forming bacteria were detected in the tip and base of the LD leaf area at 6 dpi in any of the four experiments performed.

The results suggest that plant defense responses were effective in leaves infected with relatively low densities of bacteria (including LD inoculated leaves and non-infiltrated regions of HD inoculated leaves), but plant defense capacity at the infiltration site was overcome when the bacterial density was sufficiently high.

### Photosynthesis upon Infection with *D. dadantii*

**FIGURE 3:**
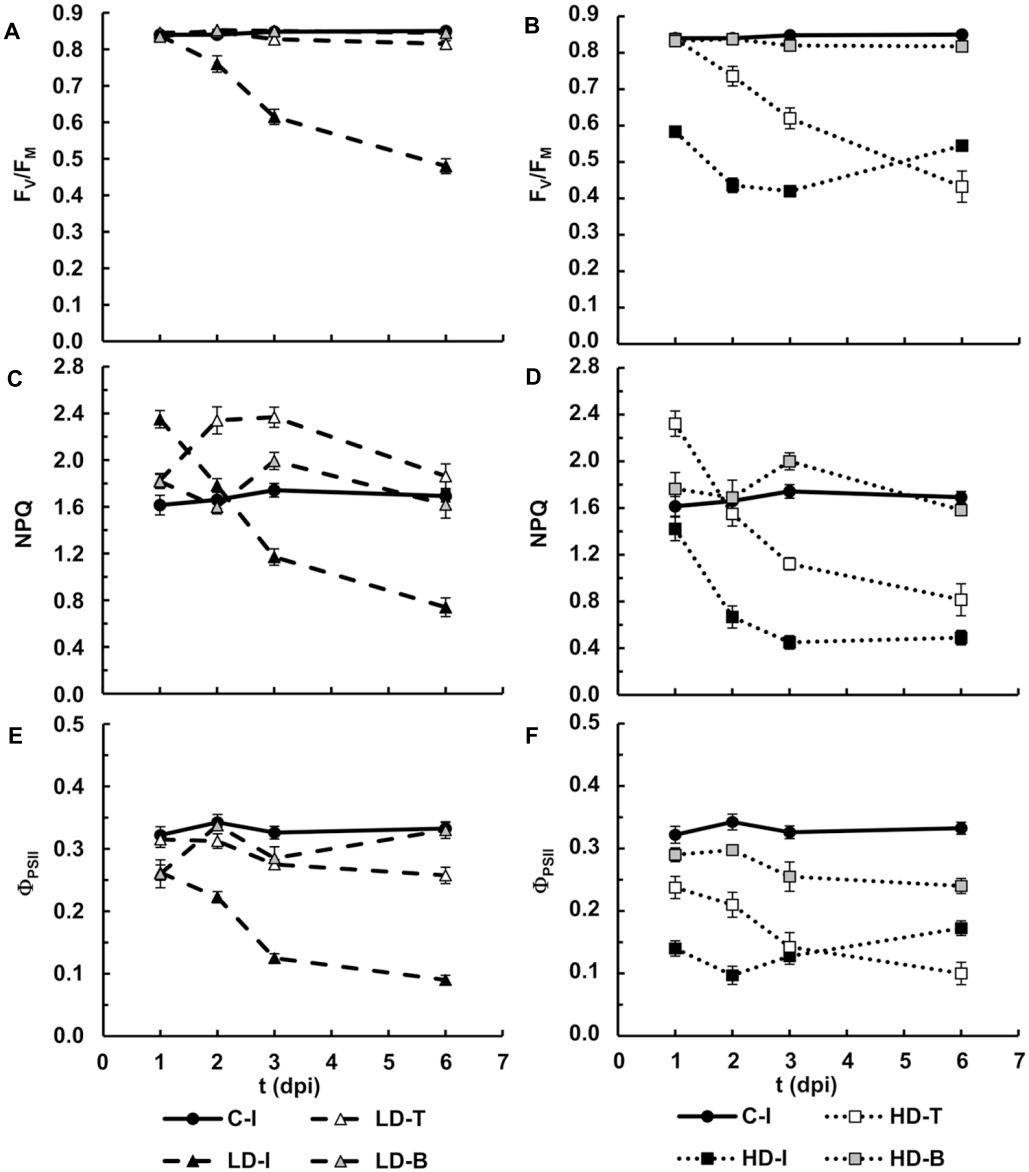
**Photosynthesis performance in *N. benthamiana* leaves inoculated with *D. dadantii***. Three regions of interest have been selected: the infiltrated (I) as well as the tip (T) and base (B) of non-infiltrated areas, surrounding the infiltrated area. Average values for maximum quantum efficiency of PSII in leaves inoculated at LD **(A)** and HD **(B)**. Average values for NPQ in the light-adapted steady state in leaves inoculated at LD **(C)** and HD **(D)**. Average values for quantum efficiency of PSII in the light-adapted steady state in leaves inoculated at LD **(E)** and HD **(F)**. Error bars mean standard error, being *n* = 6.

In LD infiltrated areas, the maximum efficiency of PSII measured as F_V_/F_M_ decreased rapidly from 2 dpi onward, indicating increasing loss of activity of PSII. There was also a very small decrease in F_V_/F_M_ in the leaf tip of the LD infiltrated leaves from 3 dpi, but no significant change in the leaf base (**Figures 1B** and **3A**). In HD inoculated areas F_V_/F_M_ was radically reduced as early as 1 dpi, indicating severe inhibition of PSII (**Figures 1B** and **3B**). Furthermore, F_V_/F_M_ in the leaf tip region of the HD infiltrated leaves decreased rapidly from 2 dpi onward, whereas the leaf base area only showed a very small decrease from 3 dpi.

A clear increase in the capacity for energy dissipation in PSII, measured as NPQ, was found in the LD infiltrated area after 1 dpi, followed by a decrease, relative to the mock-control values. In the tip of those leaves, NPQ was increased at 2 and 3 dpi and decreased to control values at 6 dpi. No significant changes were found on the base of the leaf (**Figures 1C** and **3C**). Similarly, the tip of HD infiltrated leaves showed higher NPQ at 1dpi followed by a drastic decrease and no significant differences were found on the base of the leaf. However, the NPQ in HD infiltrated areas decreased very drastically after 2 dpi (**Figures 1C** and **3D**).

Non-photochemical quenching has two contributions, reversible and irreversible NPQ. Reversible NPQ is actively controlled by the plant, regulated by the luminal pH, the xanthophylls cycle and the protein PsbS. However, the irreversible NPQ is caused by damage to the PSII. No significant differences were found in the irreversible NPQ (data not shown). Therefore, the increases found in NPQ can be attributed to changes in the capacity for reversible NPQ, positively enhanced by the plant as part of the defense response The quantum efficiency of PSII decreased progressively in LD infiltrated areas whereas HD infiltrated tissue showed no PSII activity after 1 dpi. In the non-infiltrated areas, the tip of the LD leaves showed a slight decrease in Φ_PSII_ from 3 dpi. By contrast, the decline was much more drastic in the tip and base of the HD leaves than in the corresponding areas in LD leaves (**Figures 1D** and **3E,F**).

In summary, an irreversible inhibition and loss of photosynthetic activity was found for the HD-I area from 1 dpi. The bacterial infection had the same effect in LD-I and HD-T, but 1 day delayed. On the contrary, LD-T showed an increase in the mechanisms of protection of PSII. However, regardless of the bacterial dose, the performance of photosynthesis at the base of the infiltrated leaves was little affected by the infection.

### Transpiration and Stomata Function during Infection with *D. dadantii*

**FIGURE 4:**
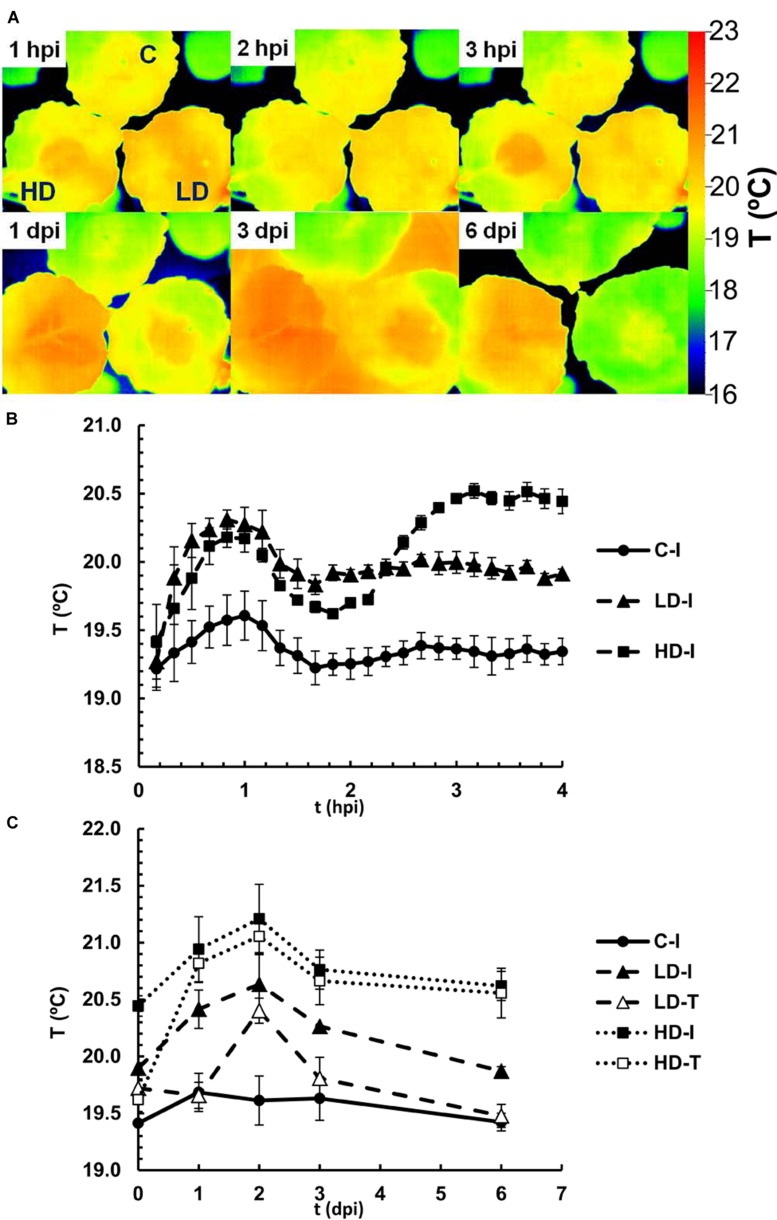
**Infrared emission of *N. benthamiana* leaves inoculated with *D. dadantii* at LD and HD, monitored by a thermal camera. (A)** False color-scale thermal images of inoculated and control leaves during the first 6 days post-infection. Images show a representative measurement. Averaged temperature values in infiltrated areas (I) and tip (T) of control and inoculated leaves during the first 4 h **(B)** or 10 days **(C)** post-infection. Data represent in both cases the average of four experiments ± standard error, *n* = 6.

**Figure 4A** shows representative thermal images of control-mock, LD and HD infiltrated *N. benthamiana* leaves during the first hours and days after inoculation. Their corresponding RGB images are shown in Supplementary Figure S1. Average temperatures were determined over the area of infiltrated and non-infiltrated regions of the leaves (**Figures 4B,C**). Thermal imaging indicated an average temperature of non-infiltrated leaves at 19.4°C (**Figures 4A,B**). During the first hour post-infiltration the temperature increased rapidly in infiltrates areas, peaking at 20.2 and 20.4°C for HD and LD treated leaves, respectively, whereas mock-inoculated areas showed a smaller increase to 19.7°C. During the next hour the temperatures decreased again and stabilized at 19.4°C in mock-inoculated and 20.0°C in LD treated leaves. In contrast, the temperature of HD infiltrated areas increased again over the next hour to 20.5°C (at 3 h post-inoculation). These temperature oscillations during the first 3 h suggest an initial stomatal closure in response to wounding and bacterial invasion, followed, in the case of mock-control and LD infiltration, by stomatal reopening. However, stomata in HD infiltrated areas closed again after 2 hpi. Over this early phase of the infection no significant differences in temperature were found for non-infiltrated regions (**Figure 4A**).

During 6 days post-infiltration, the midday temperature of infiltrated and non-infiltrated regions of control leaves remained constant at 19.5°C (**Figure 4C**). By contrast, leaves with LD and HD bacterial treatments displayed substantial increases in temperature during the first 2 days post-infiltration before leaf temperatures decreased again. In LD treated leaves, the temperature in the infiltrated region peaked at 20.6°C at 2 dpi, and then decreased slowly during the next days (down to 19.7°C at 10 dpi). In the tip of the LD infiltrated leaves, the increase in temperature was delayed, and reached a maximum of 20.4°C at 2 dpi. Furthermore, the temperature in this region decreased down to the values found for mock-control infiltrated areas after 4 dpi, suggesting a more rapid recovery. In HD treated leaves, the temperature in the infiltrated region peaked at 21.2°C at 2 dpi, and then decreased slowly during the next days. In the tip of the HD treated leaves the increase in temperature was delayed by 1 day, and thereafter followed the same trend as the infiltrated region. The high temperature in infiltrated and tip areas of HD treated leaves represent the death of the tissue and a strong decrease in the stomatal aperture in those regions, respectively.

### Secondary Metabolism in *D. dadantii*-Infected Plants

#### Localization of Secondary Metabolites *in vivo* by Imaging Techniques

*Dickeya dadantii*-inoculated plants showed higher levels of blue and green fluorescence emission (F440 and F520, respectively) than the mock-inoculated controls early in the infection process (**Figures 1E,F**). F440 increased in areas infiltrated at LD from 2 dpi, relative to the control, while in the surrounding tissues it was increased from 3 dpi. In the case of HD, F440 was slightly increased in infiltrated areas at 1 dpi. After 2 dpi, the tip of the leaf showed an increase in F440, which spread toward the base of the leaf along the infection. Changes in the intensity of F520 were larger than F440 and detected at earlier times (at 1 and 2 dpi for HD and LD inoculation, respectively). Control leaves did not show any changes in F440 or F520 during the period of study.

**FIGURE 5:**
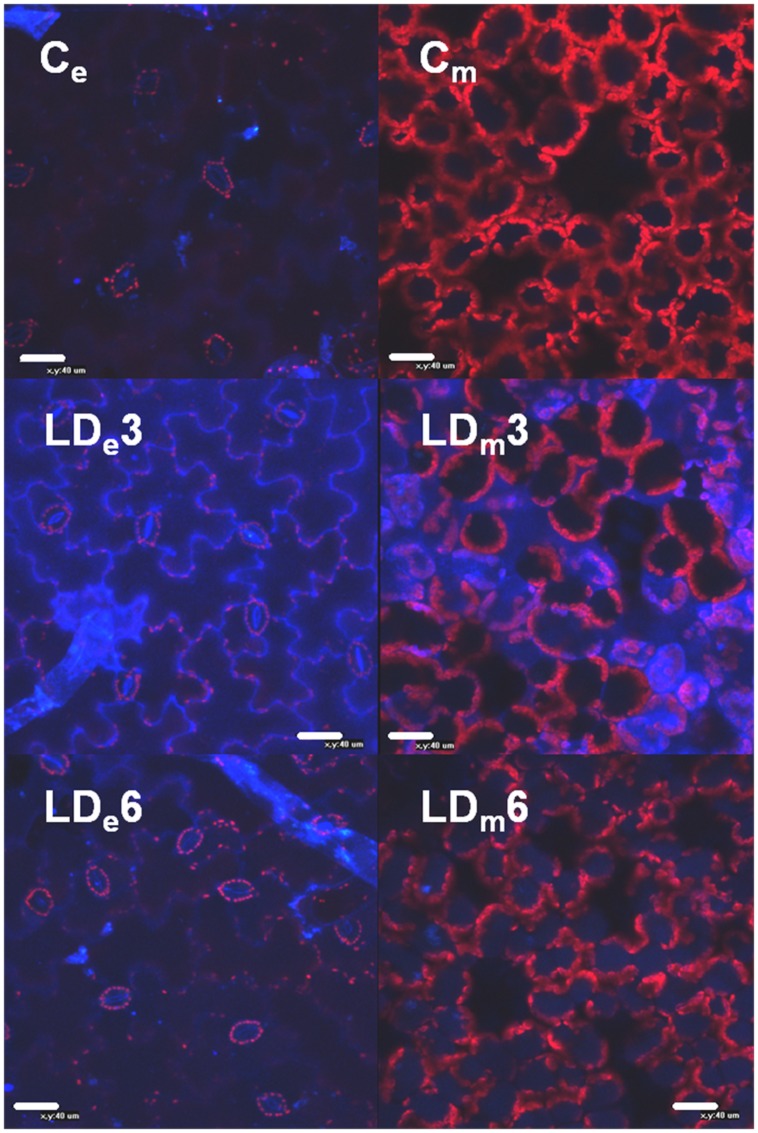
**Confocal epifluorescence micrographs of epidermal and mesophyll layers of infiltrated areas of *N. benthamiana* leaves mock-control (Ce and Cm, respectively) and infected with *D. dadantii* at LD (LDe and LDm, respectively)**. Micrographs were obtained at 3 dpi (LDe3, LDm3) and 6 dpi (LDe6, LDm6).

The source of these autofluorescence signals were localized within the leaf structure by CLSM (**Figure 5** and Supplementary Figure S2). The HD infiltrated areas quickly showed maceration and disruption of the cell structures, and displayed a very strong increase in autofluorescence emitted by phenolic compounds in the epidermis as well as mesophyll areas. In non-necrotic leaf tissues (HD-T_e_, LD-I_e_, and T_e_), autofluorescence from the cell wall of the epidermis initially increased and then declined, but at different rate and magnitude depending on the treatment and the region of the leaf. In LD infiltrated areas the autofluorescence increased strongly up to 3 dpi, and then declined at 6 dpi, whereas tip of LD leaves showed a much slower and weaker response. By contrast, the tip of HD leaves showed a quicker response, with a peak in autofluorescence at 2 dpi and a decline already at 3 dpi.

In the mesophyll of non-necrotic leaf tissues, soluble phenolic compounds initially accumulated in the apoplast, and then in the vacuoles. The intensity of the autofluorescence in the apoplast of LD infiltrated tissue increased strongly up to 3 dpi, followed by a decline and significant accumulation in the vacuoles at 6 dpi (**Figure 5** and Supplementary Figure S1). Again, the tip of LD inoculated leaves displayed a similar response, while the tip of HD infiltrated leaves showed a quicker accumulation of phenolic compounds in the apoplast as well as the vacuoles.

#### Quantification of Phenolic Compounds

**FIGURE 6:**
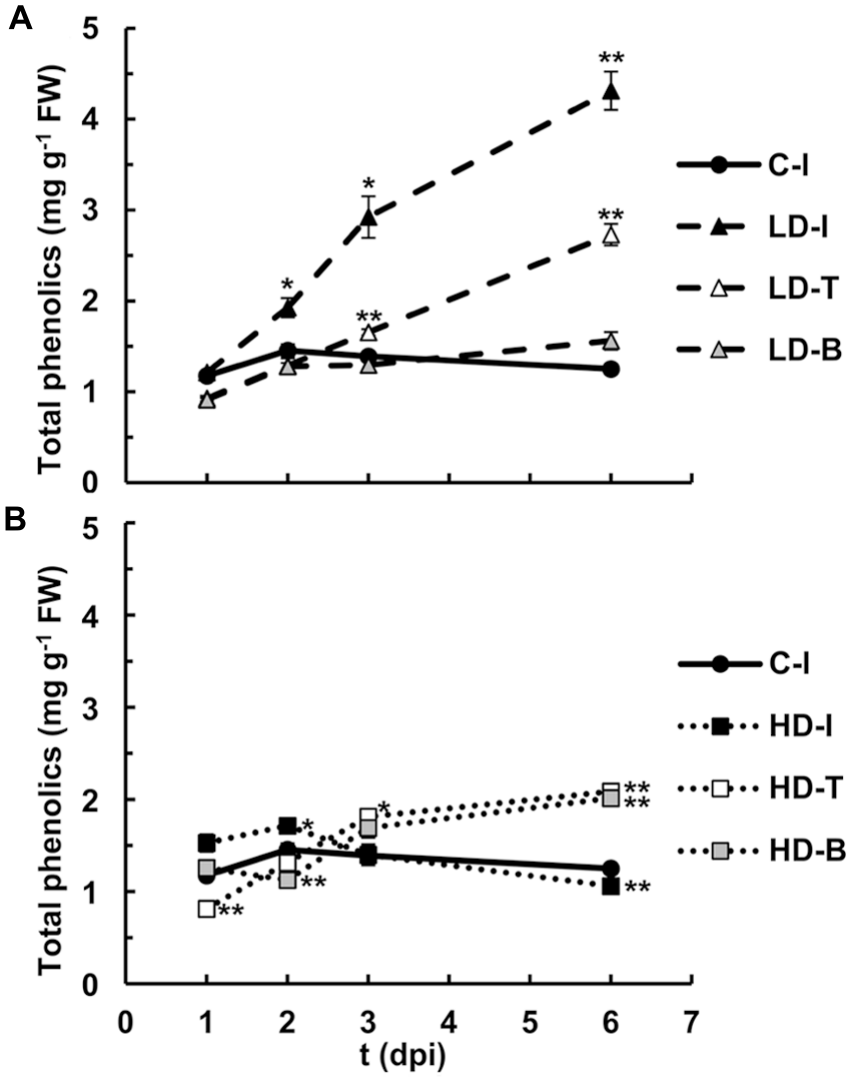
**Quantification of soluble phenolic compounds in *N. benthamiana* leaves mock-control and inoculated with *D. dadantii* at LD **(A)** and HD **(B)** for infiltrated (I), base (B) and tip (T) of the leaves**. Error bars mean standard error, *n* = 6. ^∗^*P* < 0.05; ^∗∗^*P* < 0.01.

The autofluorescence detected both in apoplast and vacuoles suggest an increase in the accumulation of soluble phenolic compounds, rather than bound to the cell walls. These compounds were quantified along the infection for infiltrated and non-infiltrated areas of the leaves (**Figure 6**). The highest contents were found in the areas infiltrated at LD from 3 dpi onward. Moreover, the tip of both LD and HD inoculated leaves accumulated soluble phenolics, reaching highest levels after 6 dpi.

**FIGURE 7:**
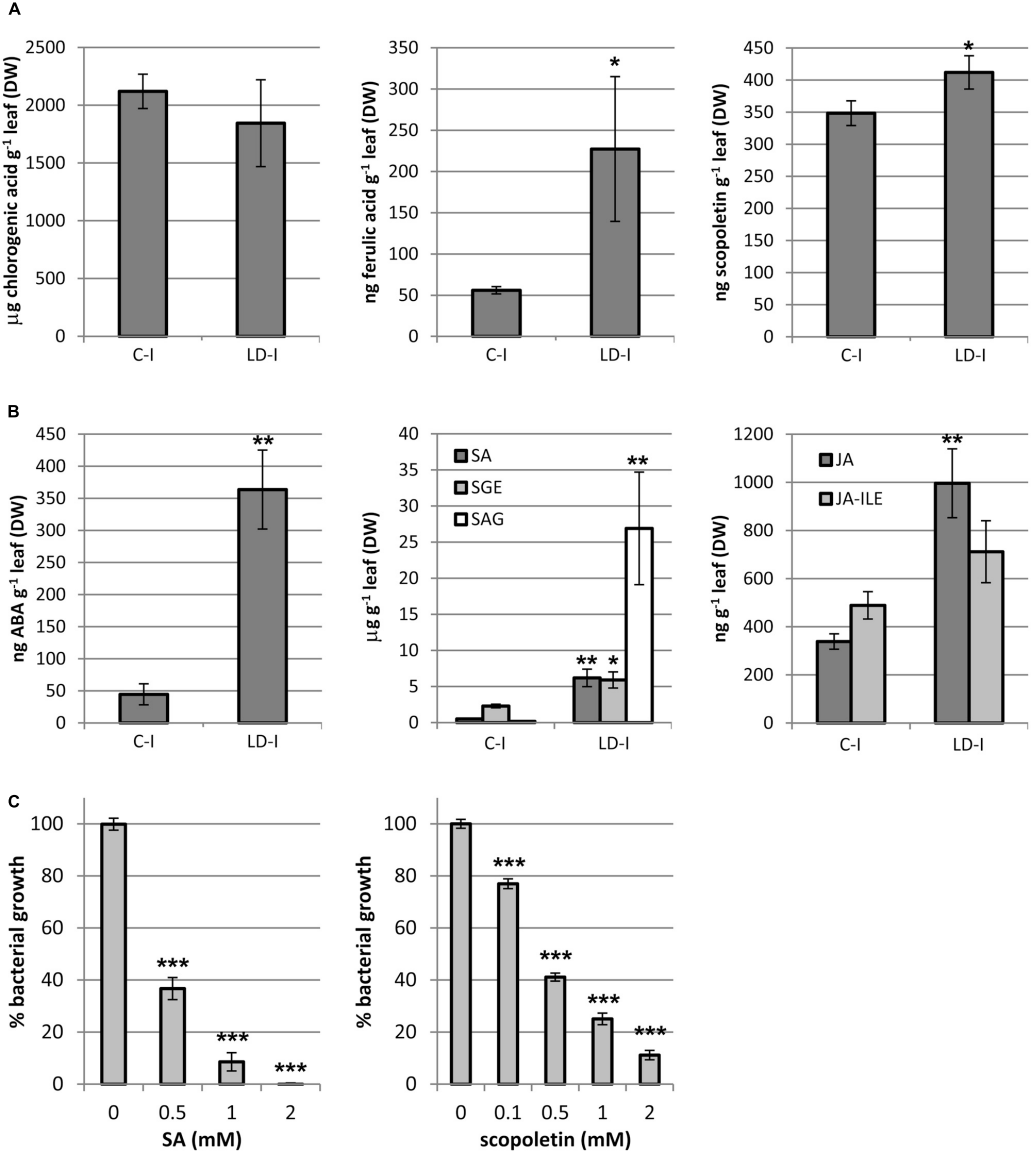
**Quantification of **(A)** phenolic compounds (chlorogenic acid, ferulic acid, and scopoletin); **(B)** hormones and its derivatives (ABA, SA, SEG, SAG, JA, and JA-ILE), in mock and LD infiltrated areas at 3 dpi. **(C)** The inhibition of bacterial growth by SA and scopoletin on *D. dadantii* is expressed as percentage of bacterial growth**. Error bars mean standard error, *n* = 5 **(A,B)**, *n* = 9 **(C)**. ^∗^*P* < 0.05; ^∗∗^*P* < 0.01; ^∗∗∗^*P* < 0.001.

Phytoalexins are soluble phenolic compounds that take part in the defense response against pathogens, and generally emit blue and green fluorescence. A selection of phytoalexins that could be produced by *N. benthamiana* under stress conditions was determined for mock and LD infiltrated areas at 3 dpi (**Figure 7**). Ferulic acid and scopoletin, two well-known phytoalexins, were found to be increased by 4 and 1.2-fold in the LD infiltrated areas, respectively. However, the accumulation of the phytoalexins caffeic acid and chlorogenic acid was not affected by the infection (data relative to caffeic acid not shown).

Many phytoalexins are well-known antimicrobials. **Figure 7** shows the capacity of SA and scopoletin for growth inhibition of *D. dadantii* 3937. Both compounds inhibited by 60% the bacterial growth at 0.5 mM and by 100 and 90% at 2 mM, respectively. Scopoletin inhibited bacterial growth by 25% at only 0.1 mM.

#### Quantification of Hormones Regulating the defense Response

The content on hormones related to biotic stress responses was analyzed in mock and LD infiltrated areas at 3 dpi (**Figure 7**). SA and JA are two main signaling molecules controlling plant immune reactions. The two active forms of SA, the free acid and its glucosyl ester (SEG), increased by 12 and 3-fold, respectively, whereas the storage form SAG was increased by almost 150-fold in the infected areas. On the other hand, the active species JA and the isoleucine conjugated acid (JA-ILE), taken together, increased by twofold (*P* = 0.017). ABA is an important regulator of plant defense against biotic stress which role is still controversial. In the LD infiltrated areas, ABA was increased by eightfold relative to the mock control. Cytokinins have been found to play a role in plant defense against some pathogens (Großkinsky et al. (2011), and references therein).

The content on kinetin, a cytokinin, was also analyzed, finding no significant changes relative to the mock-control (data not shown).

## Discussion

*Dickeya dadantii* is a broad host-range necrotroph which infectious cycle starts by the entry into the apoplast through stomata or wounds. Then, and according to the very recent review by Duprey et al. (2014), the bacteria would undergo an asymptomatic phase of the infection in which the bacteria would multiply without causing significant damage to the plant tissue. Once the bacteria were acclimatized to the environment and in high population levels, they would enter the symptomatic phase. At that time, a great amount of virulence factors would be secreted, including cell wall degrading enzymes. This transition would be triggered by the scarcity of nutrients through quorum sensing, as reviewed by Fatima and Senthil-Kumar (2015).

*Nicotiana benthamiana* was able to activate resistance mechanisms against *D. dadantii* when infiltrated at low bacterial dose, condition closer to infections in nature. And, although *D. dadantii* could overcome plant defense capacity when inoculated at high enough doses, it is remarkable that, even then, maceration was always restricted to the infiltrated area.

To analyze such plant defense mechanisms, the combination of several imaging techniques, giving temporal and spatial information about metabolic processes, have been proved to be a suitable tool. Moreover, they are useful for the detection of the infection even prior to the development of symptoms.

### Stomata take Part in Plant defense

Subsequently the first hour after infection, in which temperature of infiltrated areas could be controlled by a wounding response due to the physical damage caused during the infiltration [Desikan et al. (2002) and references therein], changes in temperature seemed to be dose-dependent. Moreover, temperature decreased down to mock-controlled levels in non-infiltrated areas of LD treated leaves parallel to the decrease in cfu. The activation of stomatal closure triggered by recognition of PAMPs in guard-cells is a widespread defense mechanism in vascular plants against invasion by the potentially vast number of bacteria to which plants are exposed in nature (Melotto et al., 2006, 2008). In this case the stomatal closure would be triggered by the detection of PAMPs and controlled by ABA via SA signaling. This is in agreement with the increase in the accumulation of ABA, SA and its derivatives here reported.

### Contribution of Photosynthesis to Plant defense

Photosynthesis supplies the plant with energy, needed for growth but also for defense. Therefore, the regulation of photosynthesis must be integrated into the defense response of plant to pathogens. The nature of photosynthetic limitations imposed by phytopathogenic bacteria is variable and they have been recently summarized by Barón et al. (2012). Bacterial challenge affects photosynthesis by both stomatal and non-stomatal limitations, causing reduction on plant CO_2_ assimilation to different extents depending on the severity and timing of infection, but also on the particular type of bacterial life style and on genotype-associated resistance of the host plant.

Several studies report the downregulation of many genes in response to bacterial infection, in particular those encoding proteins involved in photosynthesis, as recently reviewed by Bilgin et al. (2010). Particularly the decrease in the accumulation of the subunits forming the oxygen evolving complex of PSII appear to be a common feature in many plant-pathogen systems, i.e., *N. benthamiana* infected by pepper and paprika mild mottle virus (Pérez-Bueno et al., 2004), or *Arabidopsis* infected by *P. syringae* pv. *tomato* DC3000 (Rodríguez-Herva et al., 2012). The decrease in components of the oxygen evolving complex causes impairment in PSII activity and could contribute to an increase in the production of reactive oxygen species by PSII (Jones et al., 2006).

In this study *D. dadantii* caused a decrease in Φ_PSII_ accompanied by an increase in the capacity for energy dissipation or reversible NPQ (**Figure 3**), in accordance with previous reports in other plant–pathogen systems, reviewed by Berger et al. (2007). Indeed, the downregulation of photosynthesis, and therefore of PSII, has been suggested to be part of the plant defense program to limit carbon source availability for pathogens and/or to redirect carbon into secondary metabolism (Bolton, 2009). On the other hand, the protective mechanism of NPQ, which originates in PSII when downregulated, has been suggested as a positive regulator of PAMPs triggered immunity (Göhre et al., 2012). Our data correlate with this hypothesis, since the activation of the reversible NPQ is detected in the tissues in which the defense response is able to prevent maceration and/or control bacterial proliferation. Chloroplasts in LD-infiltrated areas showed a loss of functionality (very low values of all photosynthetic parameters analyzed) by 6 dpi. However, non-infiltrated areas of LD treated leaves showed control values of the photosynthetic parameters after 6 dpi, when no cfu could be detected. In the case of the HD inoculated leaves the loss of activity in the chloroplasts extended to the tip, but not to the base of the leaf.

### Role of Secondary Metabolism in Plant defense

Phytoalexins and other phenolic compounds play a crucial role in plant defense against pathogens. Indeed, several studies have correlated the accumulation levels of soluble phenolics with resistance to soft rot in potato (Kumar et al., 1991; Ngadze et al., 2012). The success of such a response depends upon the timing of their accumulation as well as the location. Accordingly, Bellow et al. (2012) showed clear differences in the location of stilbenes induced by downy mildew in different cultivars of grapevine and correlated it with their grade of susceptibility or resistance to the pathogen. These results are in accordance with the location of high autofluorescence in the cell walls of the epidermis and the apoplast of the spongy mesophyll in LD infected *N. benthamiana* tissues reported in this study (**Figure 5** and Supplementary Figure S2).

The main phytoalexins produced by *Nicotiana* sp. are phenylpropanoids and terpenoids (La Camera et al., 2004). Tobacco and *N. benthamiana* have been reported to accumulate several phytoalexins (http://solcyc.solgenomics.net/) with antimicrobial activity in response to pathogens, capsidiol and debneyol (sesquiterpenes), scopoletin (a coumarin) and (2S)-pinocembrin (a flavanone; Watson et al., 1985; Großkinsky et al., 2011). Among these compounds, which biosynthesis is activated by JA (De Geyter et al., 2012), scopoletin and its precursor ferulic acid could contribute to the inhibition of the bacterial growth. Although scopoletin increased by only 20% in the infected areas, it inhibited bacterial growth at low concentrations (**Figure 7**). On the other hand, ferulic acid is known to have antibacterial activity against *D. dadantii*, as reported by Hassan and Hugouvieux-Cotte-Pattat (2011) and confirmed by our own results (data not shown). On the other hand, SA can play a non-hormonal role in the plant (El-Mougy, 2002; Cameron and Zaton, 2004) by accumulation in the apoplast where, according to our results, it could have an antimicrobial activity against *D. dadantii* 3937.

Hydroxycinnamic acids, such as caffeic or chlorogenic acid, were found to inhibit growth of *Erwinia carotovora in vitro* (Lyon and McGill, 1988) and they accumulate in potato varieties resistant to soft rot (Thipathi and Verma, 1975). It is worth noticing here that *N. benthamiana* accumulates high levels of chlorogenic acid bound to the cell walls in response to viral infection (Pineda et al., 2008). However, the infection with *D. dadantii* did not cause an increase in the accumulation of chlorogenic acid, indicating that the production of this phytoalexin is not a general response to stress in *N. benthamiana*, but rather dependent on the type of pathogen.

The infection by *D. dadantii* activates the synthesis of ABA, SA and JA in *N. benthamiana*. ABA is known to activate the defense response against *E. carotovora* sp. *carotovora* (Ton et al., 2009). In an early phase of the infection, the accumulation of ABA is triggered by PAMPs recognition, leading to SA-dependent stomatal closure (Melotto et al., 2006). PAMPs also lead to activation of mechanisms of photoprotection such as NPQ (Göhre et al., 2012) in order to inhibit photosynthesis and to divert carbon flux into secondary metabolism (Bolton, 2009). Based on the mechanistic model suggested by Ton et al. (2009), in a later phase of the infection ABA could: (i) inhibit the SA signaling pathway (explaining the large increase of the SA pool size, but being most of it found as inactive form); (ii) and activate the JA-inducible defenses, independent of the jasmonic acid/ethylene (JA/ET) pathway, which in turn activates the ABA signaling pathway, and JA-dependent functions. The JA signaling pathway induces the expression of PAL, among others. The increase in PAL activity leads to an increase in the synthesis of phenolic compounds such as flavonoids, sesquiterpens, and lignins. In particular, ferulic acid, scopoletin and lignins are accumulated and would contribute to an increase in blue and green fluorescence (Sharan et al., 1998; Kazan and Manners, 2009; De Geyter et al., 2012; Sun et al., 2014). Moreover, scopoletin, has been found to accumulate in response to JA and responsible for an increase in the blue fluorescence, in other *Nicotiana* species (Sharan et al., 1998; Sun et al., 2014). All these findings would be in agreement with the data here reported.

Altogether, the inhibition of photosynthesis and the activation of secondary metabolism of *N. benthamiana* confer resistance to *D. dadantii* when inoculated at low bacterial dose (closer to natural infection conditions than HD). The adjustments in secondary metabolism contribute to the inhibition of bacterial growth and infectious cycle at different levels: by inhibiting maceration and therefore limiting nutrients availability to bacteria, and by inhibiting bacterial growth thanks to the production of phytoalexins.

## Author Contributions

MP-B carried out experimental design, experimental work, data analysis, and writing manuscript. EG, MP and VF carried out experimental work and data analysis. PR-P, EL-S, and MB contributed to the experimental design, writing manuscript.

## Conflict of Interest Statement

The authors declare that the research was conducted in the absence of any commercial or financial relationships that could be construed as a potential conflict of interest.

## Supplementary Material

FIGURE S1 **| RGB images corresponding to the leaves shown in **Figure 4A****.

FIGURE S2 **| Confocal epifluorescence micrographs of epidermal and mesophyll layers of control leaf, infiltrated area and leaf tip (C-Ie, C-Im, C-Te, and C-Tm); epidermal and mesophyll layers of high-density infected leaves, infiltrated areas and leaf tips, at 1, 2, and 3 dpi (HD-Ie1-3, HD-Im1-3, HD-Te1-3, and HD-Tm1-3); and epidermal and mesophyll layers of low-density infected leaves, infiltrated areas and leaf tips, at 1, 2, 3, and 6 dpi (LD-Ie1-6, LD-Im1-6, LD-Te1-6, and LD-Tm1-6)**.

## ABBREVIATIONS

ABA: abscisic acid
B: base of the leaf
cfu: colony forming units
Chl-F: chlorophyll fluorescence
CLSM: confocal laser scanning microscopy
dpi: days post-inoculation
ET: ethylene
F_0_: fluorescence in the dark-adapted state
F440: blue fluorescence emission
F520: green fluorescence emission
F_M_: maximum fluorescence in the dark-adapted state
Φ_PSII_: quantum yield of photosystem II in the light-adapted state
F_V_/F_M_: maximum quantum yield of photosystem II
HD: high dose
hpi: hours post-inoculation
I: infiltrated
JA: jasmonic acid
JA-ILE: isoleucine-conjugated jasmonic acid
LD: low dose
NPQ: non-photochemical quenching
PAL: phenyl ammonia lyase
PAMPs: pathogen-associated molecular patterns
PSII: photosystem II
SA: salicylic acid
SAG: salicylic acid β-glucoside
SEG: salicylate β-D-glucose ester
T: tip of the leaf.
