# Supplementary figures and images for: Temporal and Spatial Resolution of Activated Plant Defense Responses in Leaves of Nicotiana benthamiana Infected with Dickeya dadantii
Source: Front Plant Sci. 2016 Jan 8;6:1209. doi: 10.3389/fpls.2015.01209 (PMC4705309; doi:10.3389/fpls.2015.01209)

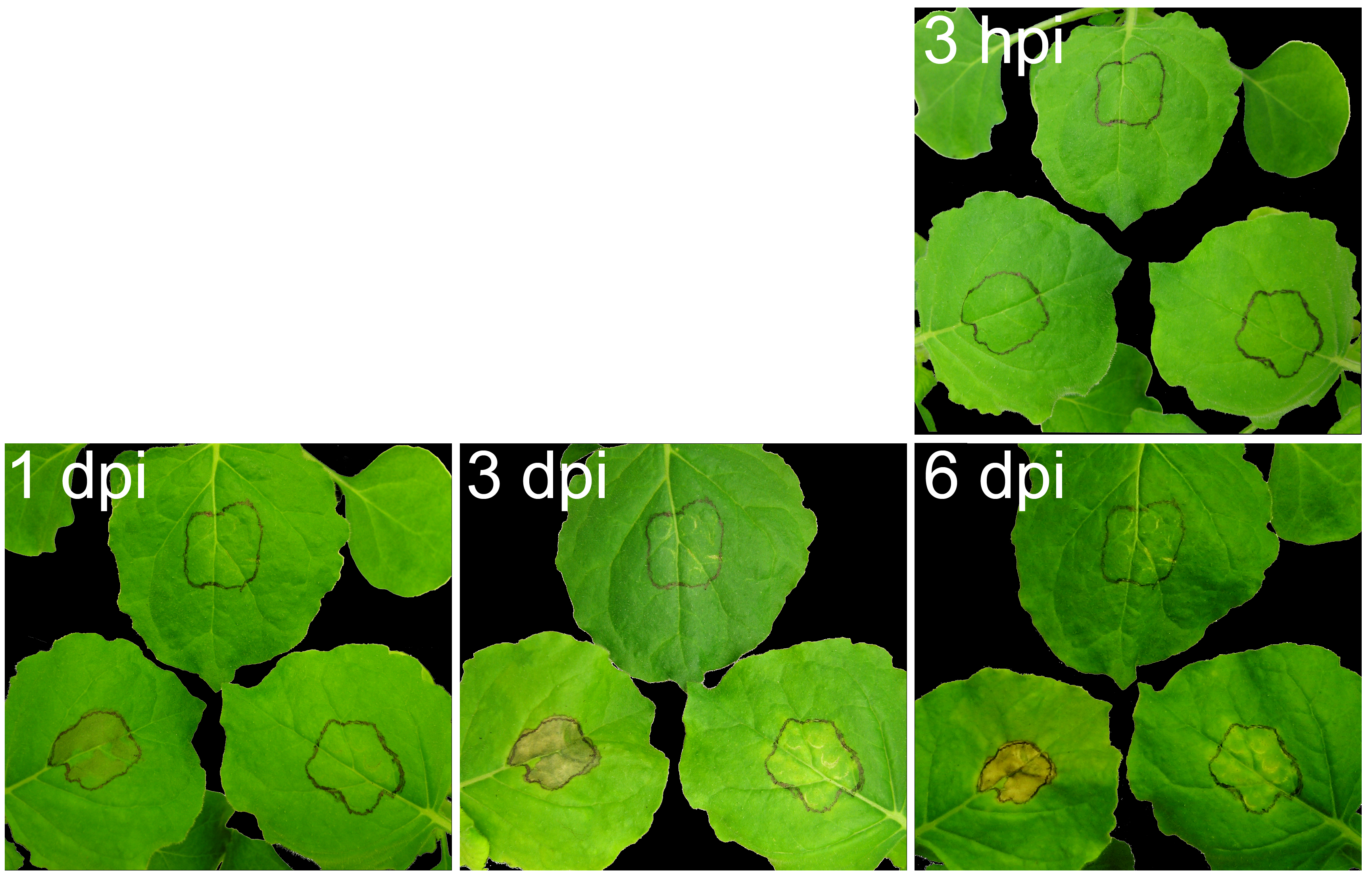

Supplement: FIGURE S1 — | RGB images corresponding to the leaves shown in Figure 4A. [file Image_1.TIF]

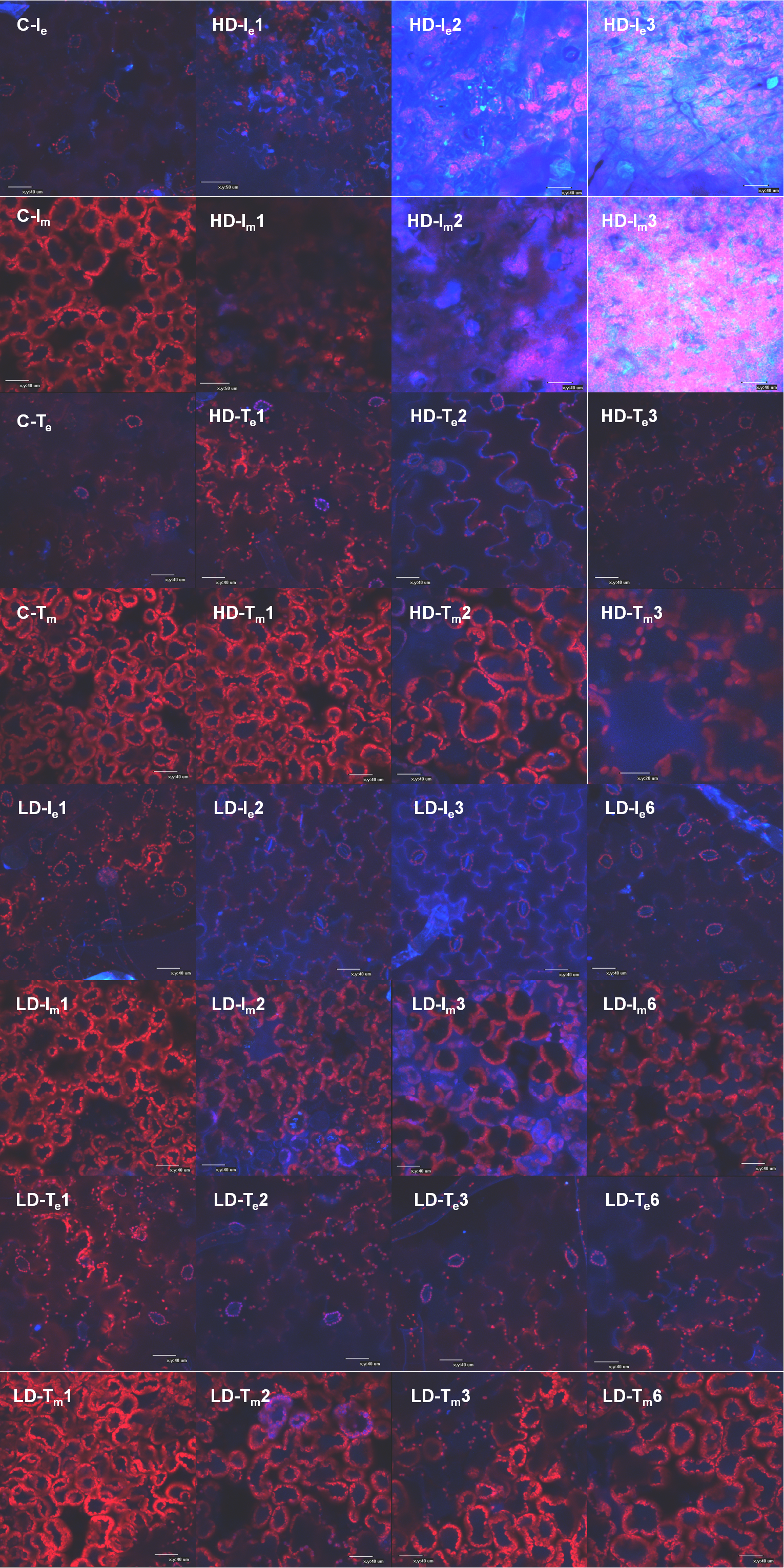

Supplement: FIGURE S2 — | Confocal epifluorescence micrographs of epidermal and mesophyll layers of control leaf, infiltrated area and leaf tip (C-Ie, C-Im, C-Te, and C-Tm); epidermal and mesophyll layers of high-density infected leaves, infiltrated areas and leaf tips, at 1, 2, and 3 dpi (HD-Ie1-3, HD-Im1-3, HD-Te1-3, and HD-Tm1-3); and epidermal and mesophyll layers of low-density infected leaves, infiltrated areas and leaf tips, at 1, 2, 3, and 6 dpi (LD-Ie1-6, LD-Im1-6, LD-Te1-6, and LD-Tm1-6). [file Image_2.TIF]
